# Changes in the fucoxanthin production and protein profiles in *Cylindrotheca closterium* in response to blue light-emitting diode light

**DOI:** 10.1186/s12934-018-0957-0

**Published:** 2018-07-09

**Authors:** Song Wang, Sujit K. Verma, Inamullah Hakeem Said, Laurenz Thomsen, Matthias S. Ullrich, Nikolai Kuhnert

**Affiliations:** 1 Department of Physics and Earth Sciences, Jacobs University Bremen, Campus Ring 1, 28759 Bremen, Germany; 2 Department of Life Science and Chemistry, Jacobs University Bremen, Campus Ring 1, 28759 Bremen, Germany

**Keywords:** Diatom, Fucoxanthin, Photobioreactor, Proteomics, Photosynthesis

## Abstract

**Background:**

Marine diatoms have a higher fucoxanthin content in comparison to macroalgae. Fucoxanthin features many potent bioactive properties, particularly anti-obesity properties. Despite the great potential for harvesting larger amounts of fucoxanthin, the impacts of light quality (light source, intensity, and photoperiod) on fucoxanthin production and the essential proteins involved in fucoxanthin biosynthesis in marine diatoms remain unclear.

**Results:**

In the present study, *Cylindrotheca closterium* was selected from four different species of diatoms based on its high fucoxanthin content and productivity. Optimal light conditions (light source, intensity, and regime) were determined by a “Design of Experiment” approach (software MODDE Pro 11 was used). The model indicated that an 18/6 light/darkness regime increased fucoxanthin productivity remarkably as opposed to a 12/12 or 24/0 regime. Eventually, blue light-emitting diode light, as an alternative to fluorescent light, at 100 μmol/m^2^/s and 18/6 light/darkness regime yielded maximum fucoxanthin productivity and minimal energy consumption. The fucoxanthin production of *C. closterium* under the predicted optimal light conditions was assessed both in bottle and bag photobioreactors (PBRs). The high fucoxanthin content (25.5 mg/g) obtained from bag PBRs demonstrated the feasibility of large-scale production. The proteomes of *C. closterium* under the most favorable and unfavorable fucoxanthin biosynthesis light/darkness regimes (18/6 and 24/0, respectively) were compared to identify the essential proteins associated with fucoxanthin accumulation by matrix-assisted laser desorption/ionization-time of flight–mass spectrometry. Six proteins that were up-regulated in the 18/6 regime but down-regulated in the 24/0 were identified as important chloroplastic proteins involved in photosynthesis, energy metabolism, and cellular processes.

**Conclusions:**

Blue light-emitting diode light at 100 μmol/m^2^/s and 18/6 light/darkness regime induced maximum fucoxanthin productivity in *C. closterium* and minimized energy consumption. The high fucoxanthin production in the bag photobioreactor under optimal light conditions demonstrated the possibility of commercialization. Proteomics suggests that fucoxanthin biosynthesis is intimately associated with the photosynthetic efficiency of the diatom, providing another technical and bioengineering outlook on fucoxanthin enhancement.

**Electronic supplementary material:**

The online version of this article (10.1186/s12934-018-0957-0) contains supplementary material, which is available to authorized users.

## Background

Diatoms (Bacillariophyta) are ubiquitously distributed in aquatic ecosystems and contribute up to 20% of the global primary production (organic compounds produced from CO_2_) [1]. Thus diatoms constitute an important autotrophic functional group in the marine food web [2]. Diatoms, besides their ecological and geochemical functions, are receiving increasing attention because of their potential use in biodiesel production [3] and for pharmaceutical purposes [4]. Furthermore, diatoms are abundant in bioactive metabolites such as antibacterial polyunsaturated fatty acids, i.e. eicosapentaenoic acid [5], and photosynthetic accessory pigments, such as fucoxanthin, both of which have been heavily investigated over the last several decades [6]. Previously, we identified and purified the benthic diatom, *Cylindrotheca closterium*, which showed outstanding characteristics in growth and lipid content [7]. Its additional characteristic of rapid sedimentation became beneficial for reducing the harvesting cost.

Fucoxanthin is a major carotenoid in diatom and brown algae. It accounts for more than one-tenth of the total carotenoid production in nature [8] and serves as a light-harvesting pigment [6]. In diatoms, fucoxanthin is primarily bound to chlorophyll a/c and forms a fucoxanthin-chlorophyll a/c protein complex (FCP), which functions as the light-harvesting complex associated with both photosystem I [9] and II [10]. Along with pigments of the xanthophyll cycle, FCPs also participate in the formation of non-photochemical quenching to avoid photo-oxidation [11]. Fucoxanthin exhibits several potent bioactivities, including anti-obesity properties, and is consequently available in various nutritional supplements [12]. The most common dietary source of fucoxanthin is brown algae in Japanese cuisine, such as in Miso soups. Commercial production of fucoxanthin mainly derives from macroalgae, such as *Laminaria japonica*, *Eisenia bicyclis*, *Undaria pinnatifida* and *Hijikia fusiformis*. These macroalgae are known to have a low fucoxanthin content [13]. Microalgae, on the other hand, with two-magnitude greater fucoxanthin content, are more promising for fucoxanthin production. Nevertheless, only a few species of marine diatoms such as *Phaeodactylum tricornutum* [14], *Odontella aurita* [13] and *Cyclotella cryptica* [15] have been studied for their commercial feasibility in fucoxanthin production.

The biosynthesis of diatom carotenoids is profoundly affected by the quality of the light [16]. Yet, the impact of light quality (light source, intensity, and photoperiod) on fucoxanthin production remains unclear. Light-emitting diode (LED) has been considered as the ideal artificial light source due to its advantages like long life-span, low heat generation, low energy consumption, and narrow light emission spectrum suited specifically for the high-value bioactive compound production [17]. Most importantly, LED light is now available at various wavelength ranges, allowing for the exploitation of photo-stimulation in natural product biosynthesis [18]. The induction of specific pigments (astaxanthin in *Haematococcus pluvialis* and β-carotene in *Dunaliella salina*) by specific LED light [19] has already been demonstrated in principle, however never applied to a natural product of commercial and medicinal interest like fucoxanthin. Red & blue and blue LED were chosen in the present experiment since fucoxanthin mainly absorbs blue light, and red mixed with blue light is beneficial for photosynthesis [6].

With more genomic and transcriptional data available from diatoms, diatom adaptation to different environments has been well understood on the molecular level. Advances in proteomics have led to a better understanding of the diatom’s proteome response to environmental changes [20]. Matrix-assisted laser desorption/ionization-time of flight–mass spectrometry (MALDI-TOF–MS) based proteomic analysis in green algae, *Haematococcus lacustris*, found proteins related to an increase of astaxanthin production under nutrient and illumination stress were up-regulated [21]. Proteomic approaches have also been used to show increased lipid biosynthesis under darkness stress [22] and high light acclimation strategy [23] in diatoms. Therefore, in our current study, a proteomic study was employed to identify the essential proteins involved in fucoxanthin biosynthesis in the diatom *C. closterium*.

*Cylindrotheca closterium* was selected from four species of diatoms as the best candidate for fucoxanthin production. An experimental model was constructed to determine the optimal light conditions (light source, intensity, and regime), leading to the highest fucoxanthin productivity while consuming the lowest amount of energy. The effects of optimal light conditions for fucoxanthin production were confirmed in bottle PBRs and, for commercialization purpose, tested in bag PBRs with continuous and flashing light. Finally, key enzymes related to fucoxanthin biosynthesis, differentially expressed under herein tested conditions, were analyzed by a proteomics approach to understand the cellular basis for the observed phenotypic changes in fucoxanthin synthesis and to predict further optimization strategies for fucoxanthin production in this model diatom.

## Results

### Screening for species with highest fucoxanthin production

**Fig. 1:**
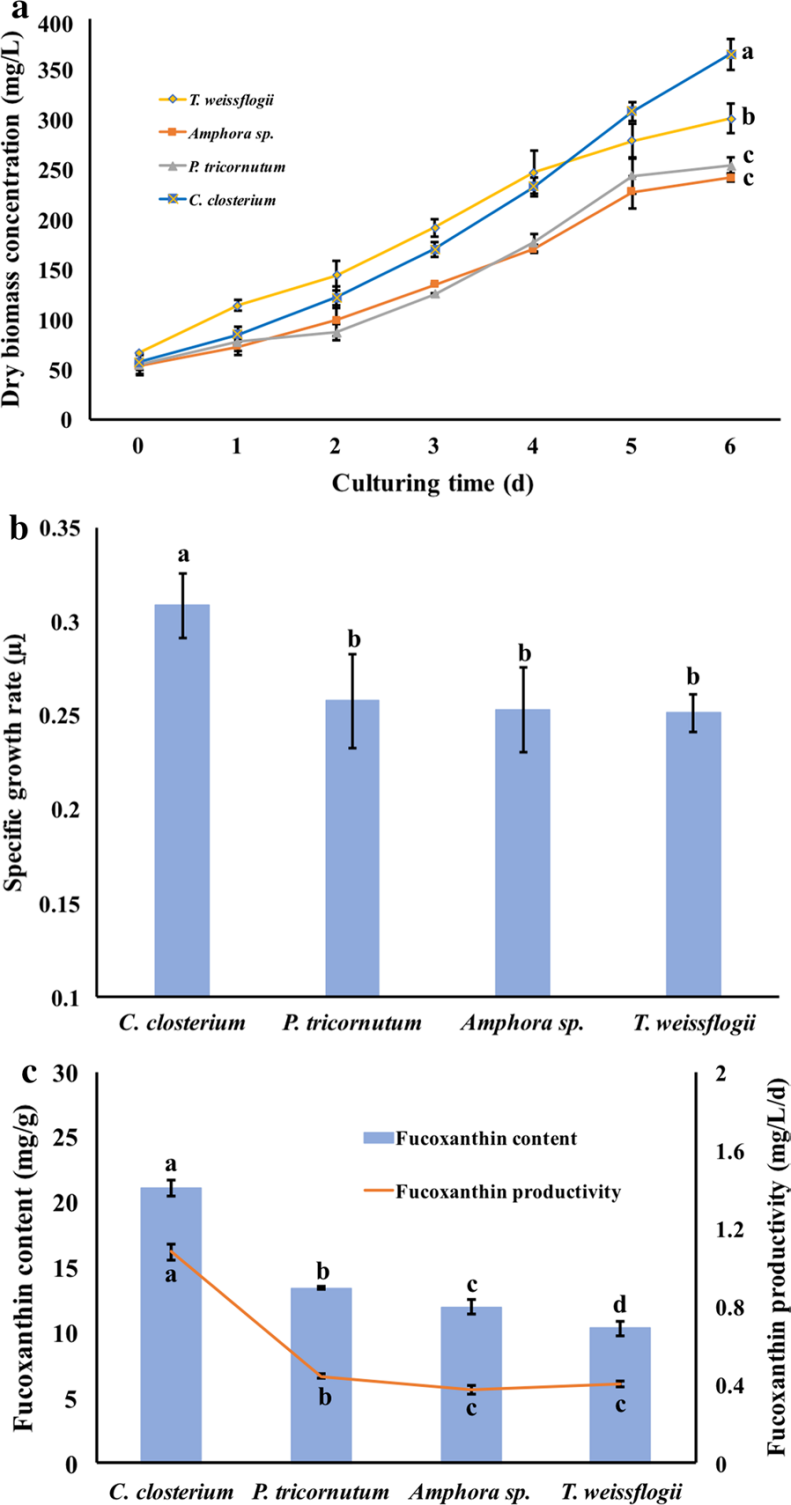
Screening of four diatom candidates. **a** Growth kinetics were monitored daily; **b** specific growth rate; **c** fucoxanthin content (column) and productivity (line) based on the dry biomass accumulation was quantified after a 6-day cultivation. The structure of fucoxanthin was inserted. Results are shown as mean ± SD, n = 3. Lowercase letters indicate statistical differences, which was analyzed by ANOVA single-factor test with an alpha value of 0.05

Four species of diatoms were cultured in bottle PBRs under the same abiotic conditions with growth monitored daily. As seen in the growth curves (Fig. 1a), *C. closterium* showed a considerable advantage in maximum biomass accumulation over the other three species of diatoms (p < 0.05). The specific growth rate of *C. closterium* (Fig. 1b) was significantly higher than that of the other three species (p < 0.05), while no significant difference was found among the other three species. *C. closterium* accumulated the highest fucoxanthin content (21 mg/g in bottle PBRs) at the end of cultivation (Fig. 1c), which is 1–2-time higher when compared to *Phaeodactylum tricornutum*, *Amphora* sp., and *Thalassiosira weissflogii*. Among all four species, *T. weissflogii* produced the lowest fucoxanthin content (10 mg/g). Notably, the fucoxanthin productivity of *C. closterium* reached as high as 1.1 mg/L/day under 12/12 light/darkness cycle, which was 2–3-time more than *P. tricornutum*, *Amphora* sp. and *T. weissflogii* (Fig. 1c). Therefore, *C. closterium* was selected to optimize the light conditions in the next step.

### Predictions by “Design of Experiment” software

**Fig. 2:**
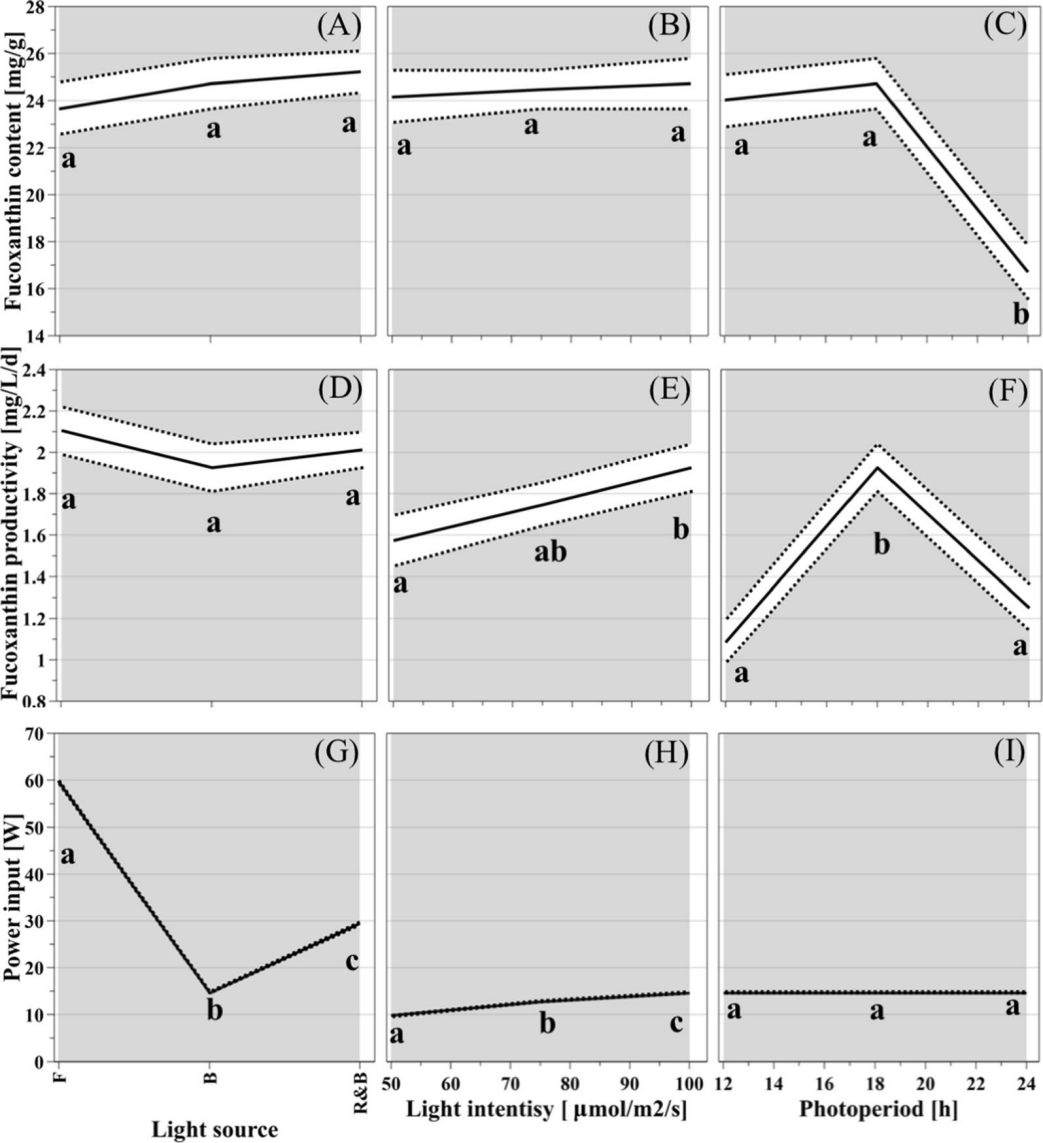
The prediction plots of “Design of Experiment”. The solid line represents the predicted value, while the dashed line represents the upper and lower limits of the confidential interval (95%). **a**, **d**, **g** The predicted responses of fucoxanthin content, fucoxanthin productivity and power input to light source, respectively, at 100 μmol/m^2^/s and 18/6 light regime; **b**, **e**, **h** the predicted responses of fucoxanthin content, fucoxanthin productivity and power input to light intensity, respectively, at 18/6 light regime with blue LED light; **c**, **f**, **i** the predicted response of fucoxanthin content, fucoxanthin productivity and power input to light regime, respectively, at 100 μmol/m^2^/s with blue LED light. Lowercase letters indicate statistical differences

**Fig. 3:**
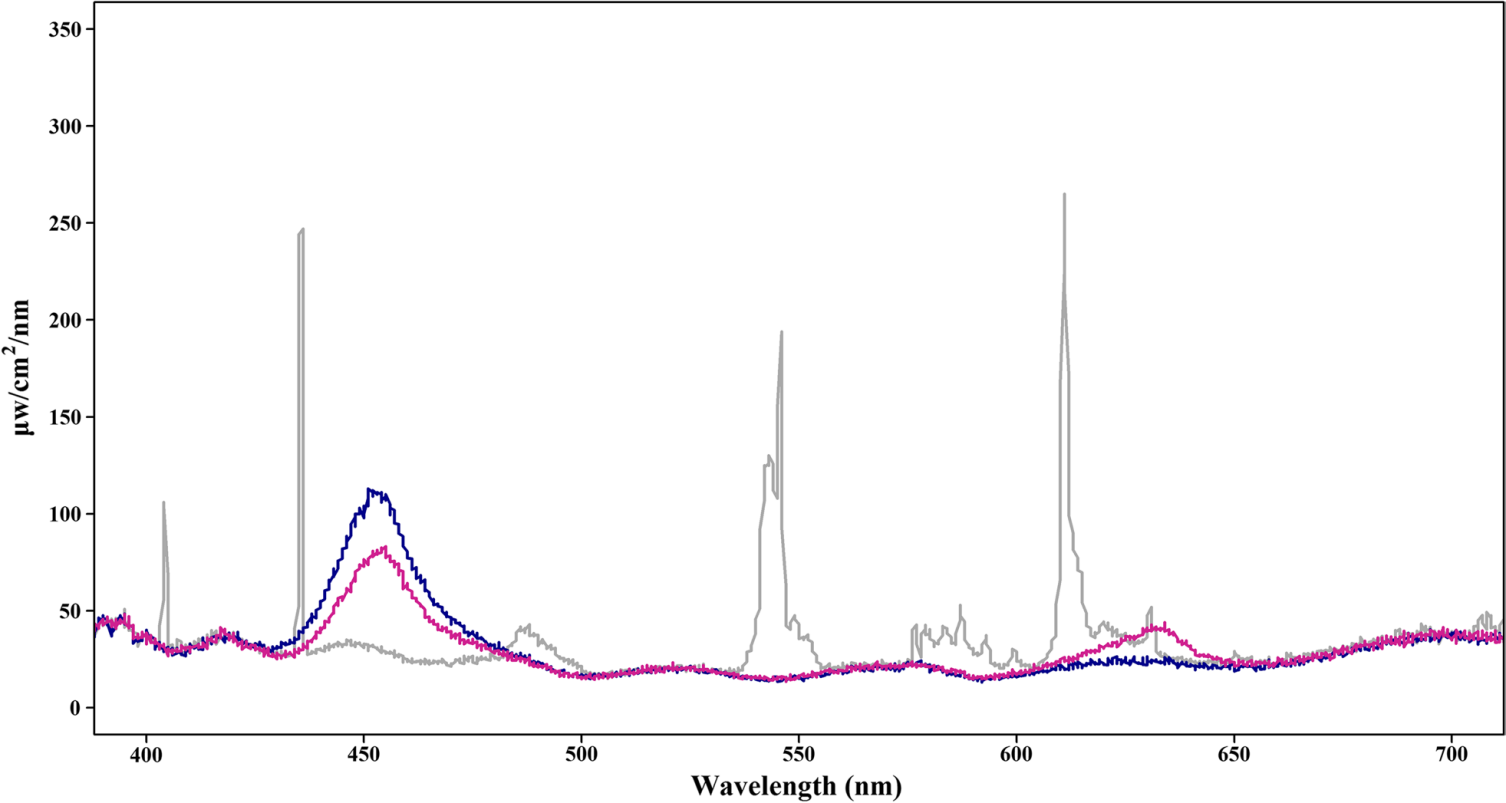
Spectra of fluorescent light, blue and red mixed with blue LED light. Grey line: fluorescent light; blue line: blue LED light; purple line: red and blue LED light

For optimization of fucoxanthin productivity in *C. closterium*, a “Design of Experiment” approach by software MODDE Pro 11 (https://umetrics.com/product/modde-pro) was chosen. Fucoxanthin content (mg/g), productivity (mg/L/day) and power input (W) were chosen as the response parameters for the predictive model. A total amount of 20 experiments led to prediction plots shown in Fig. 2 with a 95% confidence interval. When the relation between a predicted response and a light condition was discussed in each panel (a–h) individually, the other two light variables were maintained under the set constant light conditions (blue LED light at 100 μmol/m^2^/s and 18/6 light regime). To the best of our knowledge, this study, for the first time, systematically compared the use of LED light to the equivalent fluorescent light. With 95% confidence interval overlapped, no difference was found between fluorescent light and the two types of LED lights with respect to fucoxanthin content, as illustrated in Fig. 2a at 100 μmol/m^2^/s and 18/6 light regime, though different spectra of light were emitted by the three different light sources. As indicated in Fig. 3, fluorescent light (grey line in Fig. 3), compared to the two different kinds of LED light, generated broader light spectra with multiple peaks between 400–410, 430–440, 480–500, and 530–630 nm with a higher percentage of red light (600–660 nm). There is no difference found in fucoxanthin content by increasing the photosynthetic active radiation (PAR) of blue LED light from 50 to 100 μmol/m^2^/s at 18/6 light regime (Fig. 2b) with the confidence interval overlapped. A photoperiod of 24 h substantially suppresses the accumulation of fucoxanthin content with blue LED light at 100 μmol/m^2^/s, whereas 12/12 and 18/6 illumination cycles were favorable for fucoxanthin synthesis in *C. closterium* (Fig. 2c). For fucoxanthin productivity, both biomass accumulation and fucoxanthin content were taken into consideration. Fluorescent light exposure does not generate higher productivity as compared to exposures with two types of LED light (Fig. 2d) at 100 μmol/m^2^/s and 18/6 light regime. Blue LED light at 100 μmol/m^2^/s led to more fucoxanthin productivity than 50 μmol/m^2^/s but not 75 μmol/m^2^/s (Fig. 2e) under 18/6 light cycle. As indicated in Fig. 2f, blue LED light under 18/6 light regime increased fucoxanthin productivity remarkably as opposed to a 12/12 or 24/0 regime at 100 μmol/m^2^/s. A response surface plot revealing the relation between fucoxanthin productivity, the light intensity and regime of blue LED light is provided in Additional file 1: Figure S1. According to the prediction in Fig. 2g, the illumination by the blue LED light could save half or even three-fourths of the power input compared to red & blue LED or fluorescent light, respectively, at the given constant light conditions. More power (50%) was needed to generate 100 μmol/m^2^/s than 50 μmol/m^2^/s of blue LED light (Fig. 2h). Since illumination durations (72 h) were the same under different light regimes, no difference was observed in power input between different photoperiods with blue LED light (Fig. 2i). Blue LED, with one-quarter of the power input, could produce comparable fucoxanthin productivity to fluorescent light at 100 μmol/m^2^/s and an 18/6 light regime. Consequently, optimal light conditions (blue LED light at a light intensity of 100 μmol/m^2^/s and an 18/6 light/darkness cycle) were predicted to produce a maximum fucoxanthin production of 1.9 mg/L/day and a fucoxanthin content of 24.5 mg/g in dry weight under the minimum energy demand of 0.055 kWh/L/day.

### Test for large-scale production in bag PBRs

**Fig. 4:**
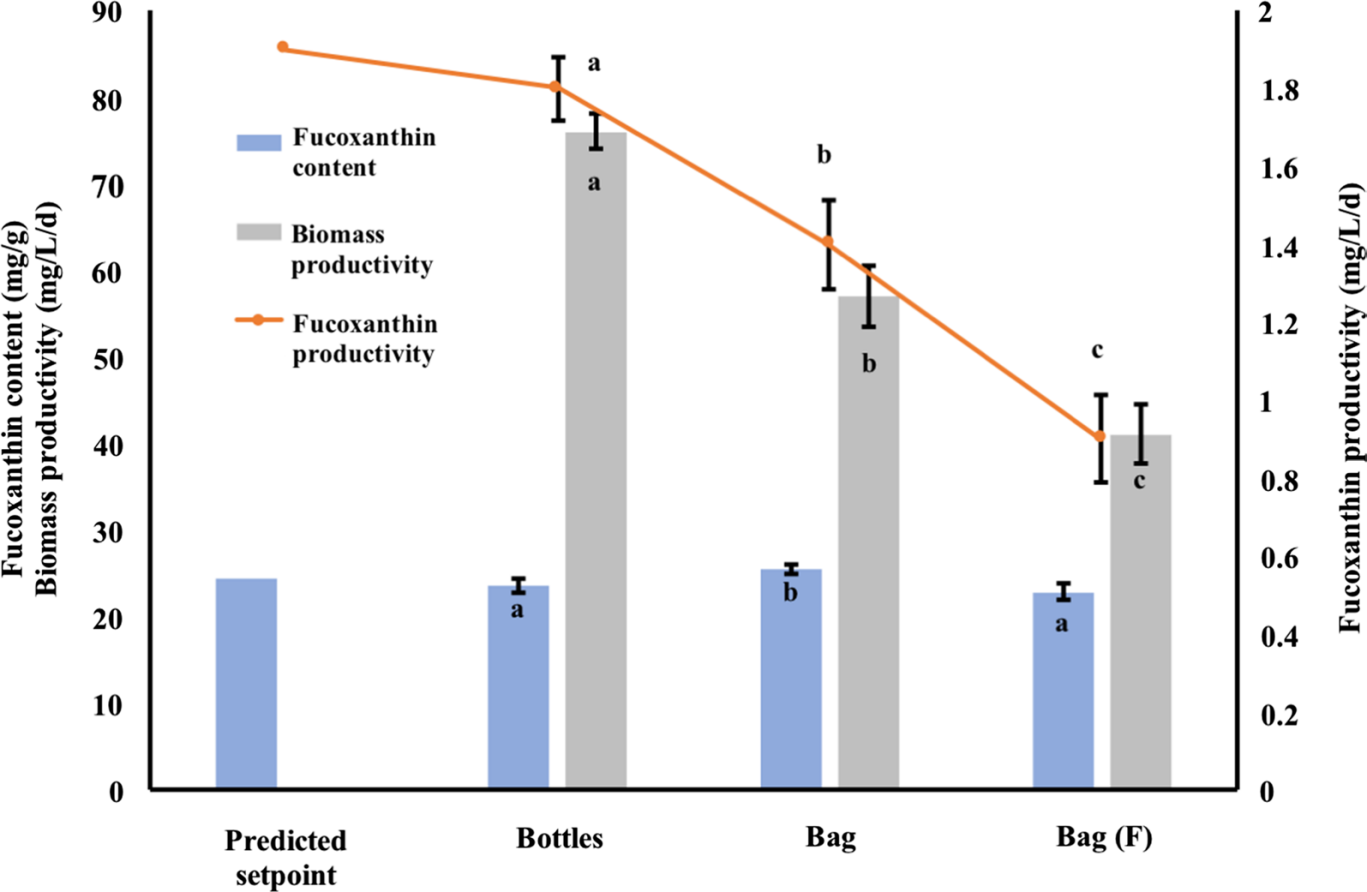
Confirmation of optimal light condition in bottle and bag PBRs. Fucoxanthin content and biomass productivity are represented by columns and productivity by line. Data was shown in mean ± SD, n = 3. Lowercase letters indicate statistical differences, which was analyzed by an ANOVA single-factor test with an alpha value of 0.05

The predicted outcome of the optimal combination of light variables was validated in the bottle PBRs and bag PBRs (Fig. 4). The biomass productivity during 4 days cultivation was found to be 76 mg/L/day in bottle PBRs versus 57 and 41 mg/L/day in bag PBRs under constant light and flashing light, respectively. This decreased biomass productivity in the bags PBRs may have been caused by a lower ratio of the aeration flow rate versus volume and light penetration rate of blue LED light in bag PBRs. A fucoxanthin content of 23.6 mg/g and productivity of 1.8 mg/L/day was obtained in the bottle PBRs (Fig. 4). These actual values were close to the predicted values of 24.5 mg/g content and 1.9 mg/L/day productivity of fucoxanthin thus proving the reliability and validity of the experimental matrix. The fucoxanthin content and fucoxanthin productivity in bag PBRs with continuous illumination during daytime reached 25.5 mg/g (an increase of 8%) and 1.4 mg/L/day (22% less), as compared to the respective values from the bottle PBRs (Fig. 4). Flashing light cultivation mode only needed half the energy consumption of continuous light cultivation, however, a noticeable decrease was observed in fucoxanthin productivity, which only reached 64% of the fucoxanthin productivity in continuous illumination (Fig. 4).

### Protein content and sodium dodecyl sulfate–polyacrylamide gel electrophoresis analyses using sodium dodecyl sulphate–dithiothreitol buffer

In diatoms, fucoxanthin is primarily bound to proteins to form an FCP complex, and it would be scientifically interesting to investigate the changes in protein profiles in response to light period changes. A proteomics study was conducted to get a deeper insight and hence, proteins were extracted from algal biomass. To obtain optimal amounts of cellular protein extracts, several methods were compared. The determined values of proteins extracted from *C. closterium* using conventional methods are shown in Additional file 1: Figure S2. Different extraction buffers led to different yields of solubilized proteins with the sodium dodecyl sulphate–dithiothreitol (SDS-DTT) buffer extraction yielding 2.5- to 5-time more extracted proteins as compared to the Tris buffer-based, pH 8.0 (buffer control) and the water-based (control) extraction methods (Additional file 1: Figure S2). Additionally, the yield of the SDS-DTT buffer extraction showed a 1.5- to 2-time rise as compared to the yield obtained by the methods using homogenization or ultra-sonication (Additional file 1: Figure S2). SDS-DTT buffer and the chemical method showed similar extractability of the solubilized proteins from *C. closterium*. However, we proceeded with SDS-DTT buffer as it yielded better protein extractability and already had been established successfully for protein extraction from higher plants such as *Theobroma cacao* [24, 25].

**Fig. 5:**
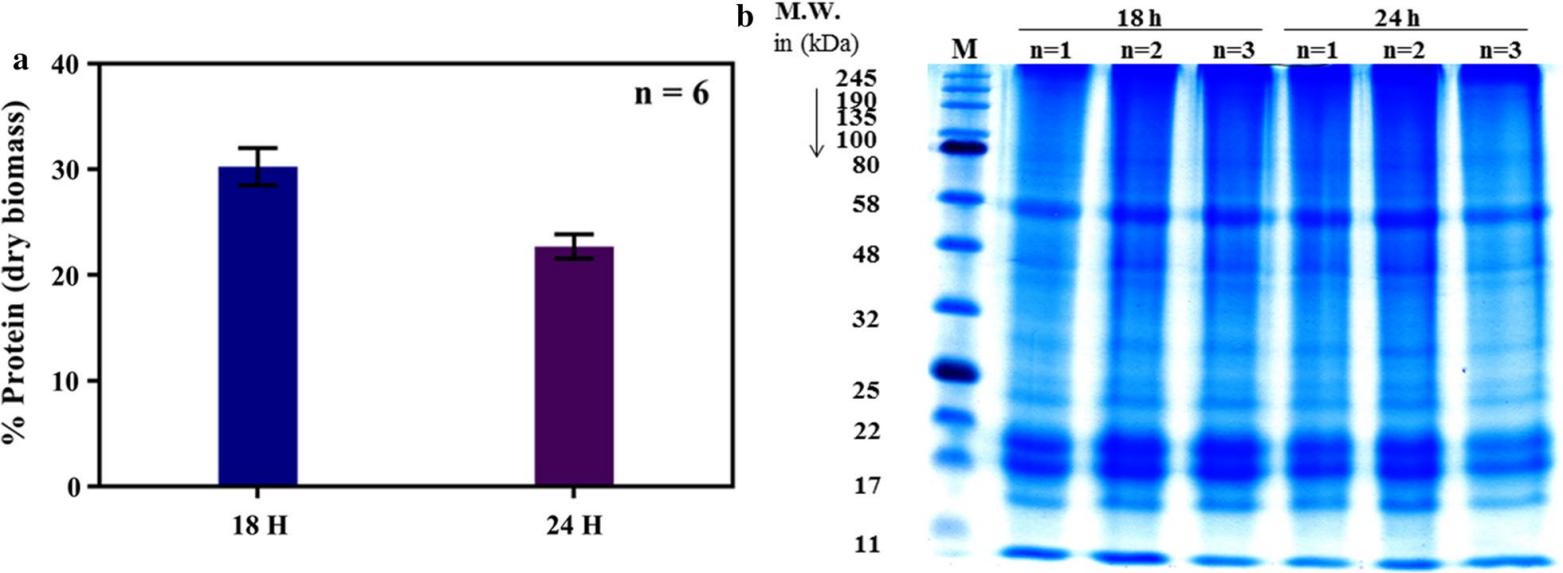
Quantification and visualization of proteins by Bradford assay and SDS-PAGE analysis. **a** Total proteins were extracted from the marine diatom *Cylindrotheca closterium* for 18 and 24 h using SDS-DTT buffer and were quantified using BCA as percentage protein of dried biomass. Bovine serum albumin was used as a standard for calibration, and the absorbance was measured at 562 nm. Measurements were done in triplicates and repeated twice. **b** The protein fractions were separated on 12.5% polyacrylamide gels and stained with Coomassie G-250. Standard protein markers were run for molecular mass determination. The three samples (n = 1, n = 2, and n = 3) were biological replicates both for the 18 and 24 h sample

To assess the overall quality of the proteins extracted from *C. closterium*, sodium dodecyl sulfate–polyacrylamide gel electrophoresis (SDS-PAGE) analysis was conducted (Fig. 5). The SDS-PAGE profiles of the extracted proteins showed high-intensity protein bands at 15, 17, 19, 25, 45, and 55-kDa (Fig. 5b). Furthermore, the SDS-PAGE analysis demonstrated that high yields of proteins could be obtained from dry biomass, and indicated that the used protein quantification data (Fig. 5a) are reliable indicators for total protein amount.

### Protein annotation via MALDI-TOF–MS analyses

**Fig. 6:**
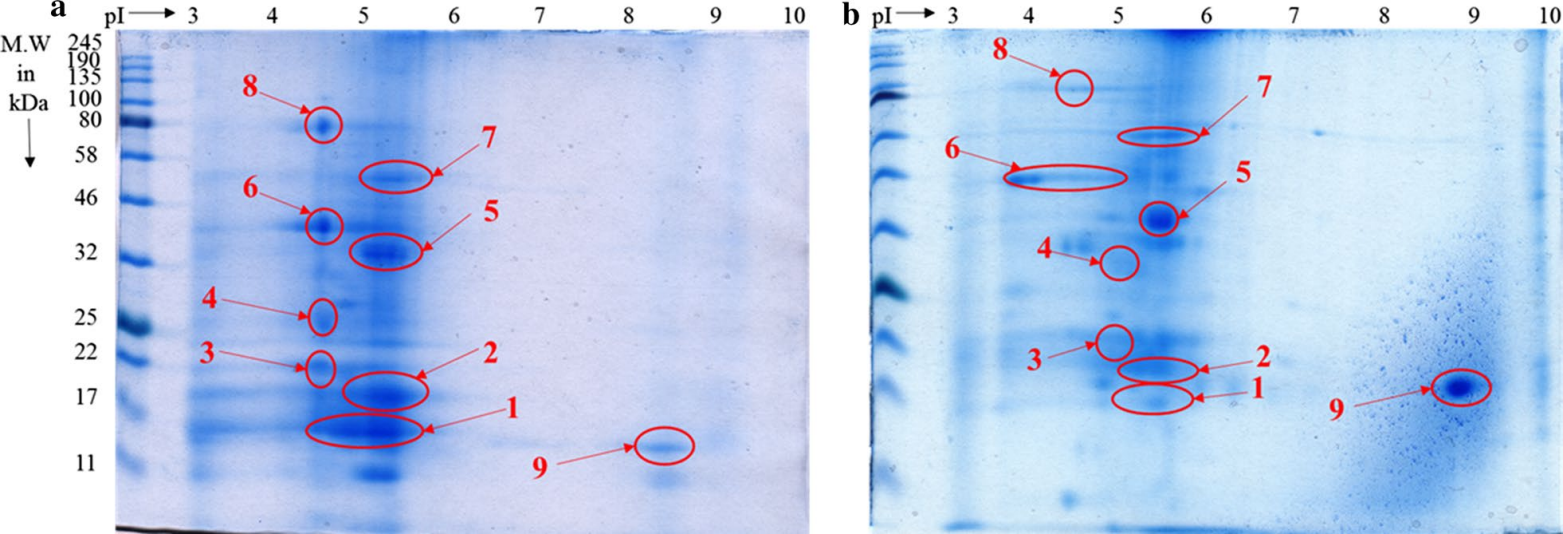
*Cylindrotheca closterium* protein visualization by two-dimensional gel electrophoresis. Total proteins were extracted from the marine diatom *Cylindrotheca closterium* from 18 h (**a**), and 24 h (**b**) dried biomass samples using SDS-DTT buffer. Sample proteins (120 μg) were loaded per gel, and standard protein markers were run on each gel for molecular mass determination. A total of 9 proteins were excised and subjected to MALDI-TOF–MS analyses, out of which only six were identified with high confidence and attributed to *C. closterium*

Proteins extracted from biomass samples from 18 and 24 h were subjected to two-dimensional polyacrylamide gel electrophoresis (2D-PAGE) analysis (Fig. 6), which separates protein both by molecular weight and isoelectric point (pI). Electrophoresis revealed well-resolved protein spots with little streaking. Most of the extracted proteins appeared to be acidic with pIs between pH 3 and 5. The 2D-PAGE of the 18 h sample (Fig. 6a) showed higher-intensity and well-resolved protein spots as compared to the 24 h sample (Fig. 6b). Subsequently, protein spot excision and proteolytic cleavage followed by MALDI-TOF mass spectrometry showed that out of the 9 well-resolved spots (marked with red circles in Fig. 6), three of the peptide mass fingerprints (spot no. 5, 8 and 9) revealed poor spectra with low intensities and could not be attributed to known protein sequences in the database. The remaining six protein spots were identified with high confidence following database searches and exhibited high intensity peptide mass fingerprint spectra attributable to proteins from the diatom *C. closterium* (Fig. 6). The protein spot numbers, matching accession numbers (NCBI and Uniprot), corresponding protein names with predicted functions and the experimental and theoretical molecular weights and pIs for each protein are summarized in Additional file 1: Table S1. All six highly expressed proteins from the two *C. closterium* cultures were critical chloroplast proteins, most of which are directly involved in photosynthesis. In terms of their predicted functions, protein spot no. 1 and 6 were assigned to the smaller and the larger subunit of the enzyme ribulose-1,5-biphosphate carboxylase/oxygenase (RuBisCO) based on their peptide fragment patterns (Additional file 1: Figure S3). Spot no. 4 was identified as the RuBisCO operon transcription regulator. Spot no. 2 and 3 were assigned to the chloroplastic ATP synthase subunits b and a, respectively. Finally, protein spot no. 7 displayed a peptide fragment pattern indicative of the ATP-dependent zinc metalloprotease, FtsH.

## Discussion

### Screening for species with the highest fucoxanthin production

It was previously reported that one strain of the *C. closterium*, cultivated in f/2 medium in the presence of antioxidant agents accumulated approximately 10 mg/g of fucoxanthin in dry biomass after a 17-day cultivation [26], which corresponds to only half of the accumulated fucoxanthin content obtained in this experiment. This difference could be attributed to the differences in the growth stage of the algal cultures during fucoxanthin collection. The fucoxanthin content of the marine diatom *Odontella aurita* decreased after reaching the late exponential phase under 100 μmol photons/m^2^/s [13]. For this reason, in our study, *C. closterium* was harvested during the late exponential phase and, as a result, synthesized remarkably higher fucoxanthin content, further highlighting the importance of harvesting time. *P. tricornutum* and *O. aurita*, exhibited high fucoxanthin productivity of approximately 0.2 mg/L/day under autotrophic cultivation [15], which by contrast is more than five times lower than the *C. closterium* culture selected in this study. Another high fucoxanthin-containing *P. tricornutum* (42.8 mg/g maximum specific fucoxanthin content) was reported previously [27], but the actual specific fucoxanthin productivity (0.64 mg/L/day) was reported to be only 60% of that of *C. closterium* from this study. Furthermore, *C. closterium,* under calm water without aeration, rapidly settles to the bottom of the bioreactor because of its benthic characteristics [28], which is beneficial for lowering dewatering costs during downstream harvesting processes. Consequently, *C. closterium* could serve as an excellent candidate for future mass fucoxanthin production.

### Predictions by “Design of Experiment” software

The application of “Design of Experiment” approach significantly increased the efficiency of experimentation. The prediction of fucoxanthin content (Fig. 2a–c) indicated no remarkable increase of fucoxanthin was induced by sole blue light at the same photosynthetic PAR and light/darkness cycle, though fucoxanthin prefers visible blue light [6]. We propose that the high light intensity caused a significant reduction of fucoxanthin content due to photo-inhibition, as was demonstrated in the marine diatom *O. aurita,* where the fucoxanthin content dropped significantly under 300 μmol/m^2^/s as compared to 100 μmol/m^2^/s light intensity [13]. This agreed with the prediction of our model, where a light intensity of 100 μmol/m^2^/s induced more fucoxanthin synthesis in *C. closterium* with blue LED light at an 18/6 regime. From the prediction of fucoxanthin productivity (Fig. 2d–f), it can be concluded that the photoperiod, not the light type nor light intensity, is the most influential parameter for optimal fucoxanthin biosynthesis in the present experimental matrix. Blue LED light, due to its low energy consumption (one-quarter of the power for fluorescent light), at 100 μmol/m^2^/s and an 18/6 light/darkness cycle was predicted to maximize fucoxanthin productivity and minimize energy consumption.

### Test for large-scale production in bag PBRs

The performance of fucoxanthin production in both bottle and bag PBRs suggested that *C. closterium* in the present study is one of the highest fucoxanthin-producing strains so far tested in bioreactors, compared to the fucoxanthin-producing algae listed in a previous study [29]. In *P. tricornutum*, fucoxanthin productivity in panel PBRs with f/2 medium was found to be 0.72 mg/L/day [27], which is half of the productivity of *C. closterium*, and 2.16 mg/L/day, in tenfold f/2 medium at 150 μmol/m^2^/s with five times more nitrogen consumption than in our study [27]. The low nitrogen consumption and energy demand (0.07 kWh/L/day including both illumination and aeration) used in our study will significantly reduce the cost of cultivation. The decrease of fucoxanthin productivity by flashing light could be that the flashing light mode is only beneficial to photosynthesis and secondary metabolites production with an excess amount of PAR to avoid the induction of photo-inhibition. PAR was not high enough to trigger severe photo-inhibition and degradation of fucoxanthin in this study. Considering that 64% of the fucoxanthin productivity was generated by using 50% energy consumption, the efficiency of light absorption was higher in flashing light cultivation. In the future, flashing LED light with a higher PAR will be tested. Ultimately, advantages such as the high expandability, low cost, ease of construction, and high energy efficiency make bag PBRs outcompete panel and column PBRs in large-scale algal plant construction.

### Protein annotation via MALDI-TOF–MS analyses

RuBisCO is a hexadecamer complex of 550 kDa, consisting of eight larger (50–55 kDa) and eight smaller (12–15 kDa) subunits [30], found in all higher plants, algae, and cyanobacteria. RuBisCO is involved in converting atmospheric carbon dioxide into energy-rich organic molecules, such as glucose, by all photosynthetic organisms [31]. In the red algae *Cyanidioschyzon merolae*, chloroplasts can autonomously activate the RuBisCO operon transcription regulator, which controls the expression of RuBisCO genes [32] in response to the activation of photosynthesis during the dark–light shift [33]. In that study, incubation of *C. merolae* for 16 h in the dark (8:16 h light/darkness) led to an increase of RuBisCO. A similar trend was observed in this study (spot no. 1 and 6) in Fig. 6. The *C. closterium* RuBisCO operon transcription regulator was expressed in the 18 h illumination sample, possibly due to a dark period of 6 h, and was down-regulated under the permanent light as seen in the form of the 25-kDa protein bands (spot no. 4) in Fig. 6.

Chloroplastic ATP synthase is involved in energy conservation in the form of ATP, and therefore is a critical component of the proton channel and plays a direct role in the translocation of protons across the membrane [34, 35]. The absence of a dark period (24 h illumination) might have led to a down-regulation of energy generation proteins such as ATP synthase, as reported previously [36] and as seen in our study, where both subunits (17 and 19 kDa) were more expressed in the 18 h illumination sample as compared to permanent illumination sample (spot no. 2 and 3) in Fig. 6. We speculate that the turnover of photosynthesis between light-dependent and light-independent phase was disturbed by the 24 h photoperiod. In the light-independent phase of photosynthesis, the down-regulation of RuBisCO and ATP synthase reduces CO_2_ fixation, NADPH consumption, and energy (in the form of ADP) turnover back to the reaction center of photo-systems in the light-dependent phase [37]. This reduction results in an ultimate overload of electron transportation and photo-oxidation in photosystems. For diatoms, it has previously been observed that the protein-bound diadinoxanthin cycle pigments participate in a mechanism of non-photochemical quenching [6].

ATP-dependent zinc metalloprotease, FtsH, belongs to the family of ATP-dependent proteases and is localized in the chloroplast [38]. Like in most photosynthetic organisms, in *Chlamydomonas* spp., FtsH was demonstrated to play a vital role in diverse protein degradation and maturation mechanisms, degradation of the photo-system (PS) II reaction center D1 protein, regulation of cytochrome b6 levels, and as a molecular chaperone involved in protein assembly [39]. The down-regulation of FtsH during permanent illumination in this study may indicate that there was an induction of photo-inhibition in *C. closterium* (spot no. 7 in Fig. 6). Since the PS II, and particularly its D1 protein, in the reaction center is subject to photo-damage, the efficiency of photosynthesis depends on the restoration of this photo-system. This repair consists of the degradation of damaged D1 protein by FtsH and re-assembly of the PS II with de-novo synthesized D1 protein [40, 41]. Consequently, a repressed repair rate of PS II during 24 h illumination could have led to a reduction of fucoxanthin content as the antenna in the form of fucoxanthin-chlorophyll a/c complexes in PS II.

In this experiment, the differently expressed proteins under an 18/6 (favorable for fucoxanthin synthesis) and a 24/0 (least favorable) light regime were determined. These differentially expressed proteins were found to be key enzymes involved in photosynthesis, but not in the de novo fucoxanthin biosynthesis. In conclusion, fucoxanthin content is intimately correlated with photosynthetic efficiency and could be further induced by an increased turnover rate between light-dependent and independent reactions. Furthermore, this work provides a novel perspective for rational, genetic engineering of fucoxanthin where future investigations could focus on the essential genes of photosynthesis, as well as the genes along the de novo fucoxanthin biosynthesis pathway.

## Conclusion

Due to its excellent growth performance and fucoxanthin productivity, the marine diatom *C. closterium*, was selected for optimizing illumination conditions using an experimental matrix design, where blue LED light, as an alternative to fluorescent light, induced maximum fucoxanthin productivity and minimum energy consumption at 100 μmol/m^2^/s and an 18/6 light/darkness cycle. Fucoxanthin productivity of 1.8 and 1.4 mg/L/day were achieved in bottle and bag PABs, respectively, with the highest fucoxanthin content of 25.5 mg/g attained in bag PBRs, which may be suitable devices for follow-up, large-scale production. Proteins induced by LED light illumination were identified by MALDI-TOF–MS. Convincingly, the up-regulation of key proteins involved in photosynthesis, energy conservation, and PS II repair mechanisms under 18 h illumination may have resulted in a higher photosynthetic efficiency as compared to permanent illumination (24 h). Our results shed valuable light on fucoxanthin regulation by photosynthesis in diatoms. Fucoxanthin, aside from the de novo biosynthesis, also correlates with photosynthetic efficiency. To further increase the fucoxanthin production, future investigations should focus on the augmentation of photosynthetic efficiency, for example, the increase of the bio-availability of CO_2_ and over-expression of the photosynthetic-related genes.

## Methods and materials

### Microalgae species and culture conditions

*Cylindrotheca closterium* and *Amphora* sp. were provided by the culture collection of the Laboratory of Applied Microalgae Biology of the Ocean University of China. *P. tricornutum* was ordered from the Culture Collection of Algae at Gottingen University. *T. weissflogii* was provided by the microbiology laboratory of Jacobs University Bremen. Stock cultures were maintained at photosynthetically active radiation (PAR) of 45 μmol/m^2^/s, 12/12 light/darkness cycle and 20 °C in f/2 medium [42]. Algae in bottle and bag PBRs were cultured in f medium.

### Equipment

Flexible LED RGB stripes with LED 5030 were purchased from LE (Hannover, Germany). Fluorescent lamps (L 58W/840) were purchased from Osram (Munich, Germany). PAR was measured with the photosynthetic yield analyzer MINI-PAM from Heinz Walz (Effeltrich, Germany). Energy consumption of illumination was measured by an energy consumption meter from Hugo Brennenstuhl GmbH (Tübingen, Germany). The spectra of fluorescent light and LED light were determined by one CCD spectrometer from Mightex (California, United States).

### Experimental design

*Cylindrotheca closterium*, *Amphora* sp., *P. tricornutum* and *T. weissflogii* were all pre-cultured to exponential phase before inoculation. Pre-cultures were diluted into bottle PBRs (in triplicates) with 800 mL of f medium to yield an initial optical density value of 0.1 at 750 nm. All cultures were kept at 20 ± 1 °C and a 12/12 light/darkness regime by fluorescent light of 80 μmol/m^2^/s. The cultures were screened for highest fucoxanthin productivity to optimize light conditions.

An experimental model regarding optimal light conditions was designed, and results were analyzed by the “Design of Experiment” software MODDE Pro 11 (Sartorius, Gottingen, Germany). The purpose of “Design of Experiment” approach was to develop a rational and efficient way to estimate the effects of variables by running the minimum number of experiments. Three illuminative variables (light source, intensity, and regime) were evaluated by response factors (fucoxanthin content, fucoxanthin productivity, and energy consumption). With the objective of optimization, a quadratic model comprised of 20 runs of experiments with three center points was built (Table 1). Algae in every treatment received of a total illumination period of 72 h before harvesting. The accuracy and validity of the predicted optimal light conditions were confirmed with experiments in bottle PBRs. Furthermore, the feasibility of large-scale production was tested in bag PBRs (patented by Phytolutions GmbH, Bremen, Germany) as a prototype (Additional file 1: Figure S4), of which the robustness and expandability had been tested in large-scale production in the field [43]. Illumination of two bag PBRs with a volume of 20 L was provided by LED plates mounted with four LED stripes lit continuously or flashing at 1 Hz with a duty cycle of 50%. The protein of *C. closterium* cultured under 18/6 or 24/0 blue LED light regime at 100 μmol/m^2^/s (with triplicates) was evaluated by a proteomics approach to potentially elucidate the mechanism of light-induced fucoxanthin biosynthesis in this diatom.

**Table 1:** Experiments recommended by “Design of Experiment” software

Experiment no.	Light intensity (μmol/m^2^/s)	Light regime (h/h light/darkness)	Light source
1	50	12/12	Fluorescent
2	100	12/12	Fluorescent
3	75	18/6	Fluorescent
4	50	24/0	Fluorescent
5	100	24/0	Fluorescent
6	50	12/12	Blue
7	100	12/12	Blue
8	75	18/6	Blue
9	50	24/0	Blue
10	100	24/0	Blue
11	50	12/12	R & B
12	75	12/12	R & B
13	100	12/12	R & B
14	50	18/6	R & B
15	100	18/6	R & B
16	50	24/0	R & B
17	75	24/0	R & B
18	75	18/6	R & B
19	75	18/6	R & B
20	75	18/6	R & B

Fluorescent: fluorescent light; blue: blue LED light; R & B: red and blue LED light. Experiment No. 18–20 are center points

### Measurement of growth kinetics, biomass productivity, and specific growth rate

The growth kinetics of the diatoms in the screening experiment were monitored daily by determining the dry biomass concentration (mg/L) with a gravimetric method. Algal suspension (30 mL) was filtered onto pre-weighed 47 mm Whatman GF/F filters (Maidstone, United Kingdom) and algae rinsed by ultrapure water to remove the salts. The obtained filters were dried in an oven for 24 h at 60 °C and then weighed subsequently to calculate the dry biomass concentration (mg/L). Biomass productivity (P) was calculated with the following equation: P = (X_t _− X_0_)/(t − t_0_) [44] and specific growth rate (µ) was calculated with the equation: µ = (ln X_t_ − ln X_0_)/(t − t_0_) [45], in which X_t_ and X_0_ represent the dry biomass concentration on time points t and t_0_ respectively. Since the inoculum for bag PBRs is cultivated in bottle PBRs, the initial concentration (X_0_) was calculated by the correlation curve between cell number and dry biomass in bottle PBRs with an R^2^ = 0.99635. Cell concentration in bottle PBRs was determined with a cell counting chamber from Paul Marienfeld GmbH (Lauda-Königshofen, Germany).

### Fucoxanthin extraction, identification, and quantification

Volumes of 30 mL algal suspension were harvested by centrifugation at 3000 ×*g* at 16 °C for 5 min. Algae biomass was rinsed twice with ultrapure water to remove seawater. Samples were stored at − 20 °C and subsequently lyophilized for 24 h. Pre-weighed samples were extracted by vortexing with sterile glass beads for 15 min in methanol. The extraction procedure was then repeated. Methanolic extracts were combined, diluted 1 to 20 and filtered through 0.2 μm pore size filters before quantification by high-performance liquid chromatography (HPLC). Every step was performed in a dim environment due to the light sensitivity of fucoxanthin. The fucoxanthin content of all samples was analyzed by a Thermo Fisher Ultimate 3000 HPLC, (Waltham, United States) equipped with a C18 reverse-phase column. The mobile phase consisted of acetonitrile, methanol, and water (70:25:5) with 50 mg/L ammonia acetate. A standard calibration curve (R^2^ = 0.9999) was made by taking five points between the range of 0.25 and 0.5 μg/mL with fucoxanthin standards dissolved in methanol (Sigma Aldrich, Darmstadt, Germany). Samples were diluted 10–20 times to the range of the calibration curve, and 50 μL of each sample was injected into the HPLC. Absorbance was recorded at 445 nm [14]. Fucoxanthin productivity was calculated by multiplying fucoxanthin content with biomass productivity.

### Protein extraction using SDS-DTT buffer

Protein extraction was performed according to a previously described method [24] with minor modifications. Dried biomass (50 µg) was incubated in 5 mL of SDS-DTT buffer for 10 min at 80 °C to activate the protein extraction and then left rotating at 40 °C for 2 h. After 2 h, the samples were centrifuged at 3220 ×*g* for 20 min at room temperature. Then the protein-containing supernatant was collected. Ice-cold acetone was used to precipitate the protein from the supernatant. The precipitate was incubated at − 20 °C overnight. On the next day, the samples were centrifuged at 16,000 ×*g* for 20 min at 4 °C in a bench top 5415R centrifuge (Eppendorf, Hamburg, Germany), and the pellet was re-suspended in 50 mM Tris–HCl buffer (pH of 8.0). The acetone precipitation of the protein was repeated twice. Subsequently, the protein pellet was either re-solubilized in 1 mL of 50 mM Tris–HCl, pH 8.0, and then directly used in SDS-PAGE analysis or was re-solubilized in 1 mL of rehydration buffer (2 M thiourea, 6 M urea, 16.2 × 10^−3^ M 3-[(3-Cholamidopropyl) dimethyl ammonio]-1-propane sulphonate, 25.9 × 10^−3^ M DTT) supplemented with ampholytes (BioRad, Munich, Germany) according to manufacturer’s specification and used for 2D-PAGE analysis.

### Determination of protein concentration

The protein content of extracted algal protein solutions was assessed using the BicinChoninic Acid (BCA) method [46]. The BCA kit was purchased from Thermo-Fischer (Schwerte, Germany). The protein concentrations were measured in triplicates according to the provided protocol.

### Sodium dodecyl sulfate–polyacrylamide gel electrophoresis

The extracted algal proteins were separated according to their molecular weight using SDS-PAGE. The protein sample (30 µg) was mixed with 6 × sample buffer (5 µL) [47] containing bromophenol blue as tracking dye. The mixture was heated at 95 °C for 5 min and loaded onto SDS–PAGE gels (83 mm × 65 mm × 1 mm) containing 12.5% (w/v) acrylamide. The SDS-PAGE gels were run in the Mini-PROTEAN Tetra cell system from BioRad. Electrophoresis was done at 130 V for 90 min. The resulting gel was stained with Coomassie^®^ Blue [45% (v/v) methanol, 10% acetic acid, 2.93 × 10^−3^ M Coomassie^®^ Brilliant Blue G-250] for 20 min and further treated with a de-staining solution [10% (v/v) acetic acid, 5% (v/v) 2-propanol] overnight with gentle shaking.

### Two-dimensional protein gel electrophoresis

Two-dimensional (2D) gel electrophoresis of algal proteins was performed by isoelectric focusing and subsequent SDS–PAGE. For this, 120 μg of protein were applied to immobilized pH gradient (IPG) strips (7 cm, pH 3-10; Bio-Rad) by soaking for 16 h at room temperature. Isoelectric focusing was carried out on a Bio-Rad Protean^®^ i12TM IEF Cell (50 V, 70 min; 150 V, 20 min; 300 V, 15 min; gradient to 600 V, 10 min; 600 V, 15 min; gradient to 1500 V, 10 min; 1500 V, 30 min; gradient to 3000 V, 20 min; 3000 V, 210 min; pause on 50 V). Next, IPG strips were equilibrated for 15 min in 6.48 × 10^−2^ M DTT and 0.216 M iodoacetamide solution dissolved in equilibration buffer [6 M urea, 30% (w/v) glycerol, 69.2 × 10^−3^ M SDS in 0.05 M Tris–HCl buffer, pH 8.8] at room temperature. Molecular weight separation was conducted on a Bio-Rad Mini-Protean^®^ Tetra System (50 mV, 10 min; 110 mV further on) via a 12.5% polyacrylamide gel. The molecular weight of the proteins was assessed by their visual mobilization in polyacrylamide gel and the predicted weight of the amino-acid sequence. The resulting gel was stained and de-stained as described above.

### In-gel protein digestion

Protein spots of interest were excised from the SDS polyacrylamide gels, chopped into small pieces, and washed twice for 15 min in 100 μL of 0.05 M ammonium bicarbonate buffer, containing 50% acetonitrile (ACN) (v/v). Gel pieces were then dehydrated by the addition of 500 μL ACN and incubated at room temperature (RT) for 10 min. After decanting and a short air-drying, samples were supplemented with trypsin digestion buffer as previously established [48]. The tryptic digest was carried out at 37 °C overnight. On the next day, the sample was directly used for MALDI-TOF–MS analyses.

### MALDI-TOF–MS analyses of proteins

For spectrometric identification of peptide patterns, 1 µL of protein digest solution was mixed with the same volume of α-Cyano-4-hydroxycinnamic acid solution (prepared in 85% ACN, 15% H_2_O, 0.1% trifluoroacetic acid and 0.001 M ammonium dihydrogen phosphate), and spotted on a MTB AnchorChip target with an anchor diameter of 600 μm (Bruker Daltonics, Bremen, Germany). Spots were left for drying followed by an additional spotting of 1 µL of 2, 5-dihydroxybenzoic acid. After further drying the samples were subsequently submitted to an Auto flex II TOF/TOF mass spectrometer (Bruker Daltonics), which was used with standard parameters [acquisition range 500–4000 Da; S/N = 6, in specific cases 3; error range 50 ppm; allowed miss cleavages = 1; potential modifications ‘Oxidation (M)’]. Peptide masses derived from trypsin auto-digestion were used for calibration (842.50940; 1045.56370; 1713.80840; 1774.89750; 2083.00960; 2211.10400; 2283.18020 Da). Raw data were processed with Flex Analysis, version 3.0 (Bruker Daltonics). Protein identification was carried out using the Mascot search engine [49], using the Bio-tools software, version 3.1 (Bruker Daltonics). Mass lists were searched against the NCBI database [50]. The restricting taxonomy frame for the search was set to “other eukaryotes” in the NCBI database, and the Mascot score probability was set at p < 0.05. Due to this setting, the significance threshold for a score was set between 70 and 80 [49]. Oxidation of methionine [‘Oxidation (M)’] was selected as variable modification. For fixed modifications, carbamidomethyl (C) was selected. The mass error for tryptic peptide identification was set at 50 ppm, and the measurements were done in positive ion mode.

### Data analysis

The significance of variance was analyzed by analysis of variance (ANOVA) single factor analysis (p < 0.05) in Microsoft Office Excel.

## Additional file

**Additional file 1: Figure S1**. Surface response plot. **Figure S2**. Quantification of proteins by Bradford assay (A). **Table S1**. Summarized table for the proteins identified. **Figure S3**. Peptide mass fingerprint mass spectra as observed by MALDI-TOF–MS analysis. **Figure S4**. Bag photobioreactors with LED illumination.

## Abbreviations

FCP: fucoxanthin-Chl a/c protein complex
LED: light-emitting diode
MALDI-TOF–MS: matrix-assisted laser desorption/ionization-time of flight–mass spectrometry
PBR: photobioreactor
PAR: photosynthetic active radiation
OD: optical density
HPLC: high-performance liquid chromatography
SDS-PAGE: sodium dodecyl sulfate–polyacrylamide gel electrophoresis
SDS-DTT: sodium dodecyl sulphate–dithiothreitol
pI: isoelectric point
RuBisCo: ribulose-1,5-biphosphate carboxylase/oxygenase
PS: photosystem
BCA: bicinchoninic acid
ANOVA: analysis of variance
