# Supplementary material for: Changes in the fucoxanthin production and protein profiles in Cylindrotheca closterium in response to blue light-emitting diode light
Source: Microb Cell Fact. 2018 Jul 9;17:110. doi: 10.1186/s12934-018-0957-0 (PMC6036692; doi:10.1186/s12934-018-0957-0)
Supplement: Supplementary file 1 — Additional file 1: Figure S1. Surface response plot. Figure S2. Quantification of proteins by Bradford assay (A). Table S1. Summarized table for the proteins identified. Figure S3. Peptide mass fingerprint mass spectra as observed by MALDI-TOF–MS analysis. Figure S4. Bag photobioreactors with LED illumination. [file 12934_2018_957_MOESM1_ESM.pdf]

Supplementary materials

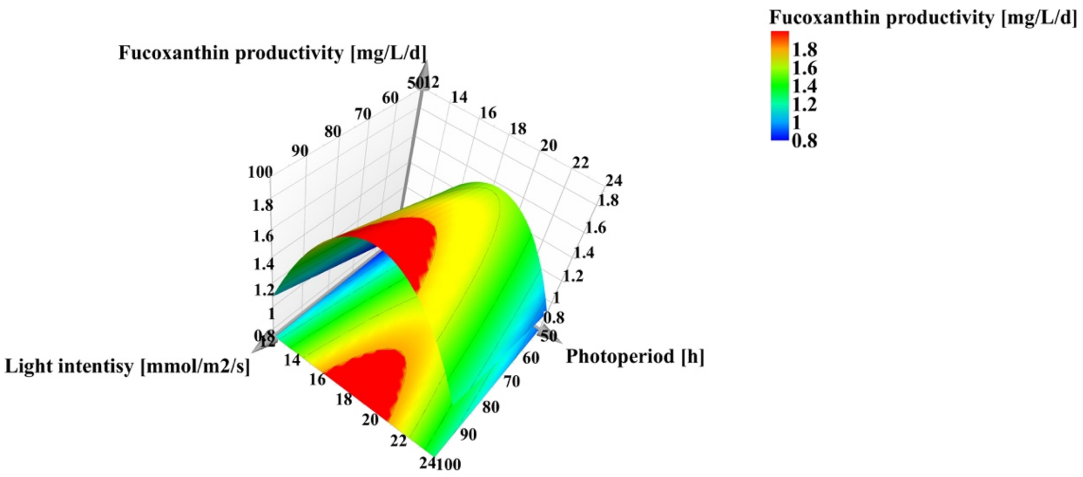

Figure S1. Surface response plot. It indicates relation between fucoxanthin productivity, light intensity and photoperiod with blue LED light.

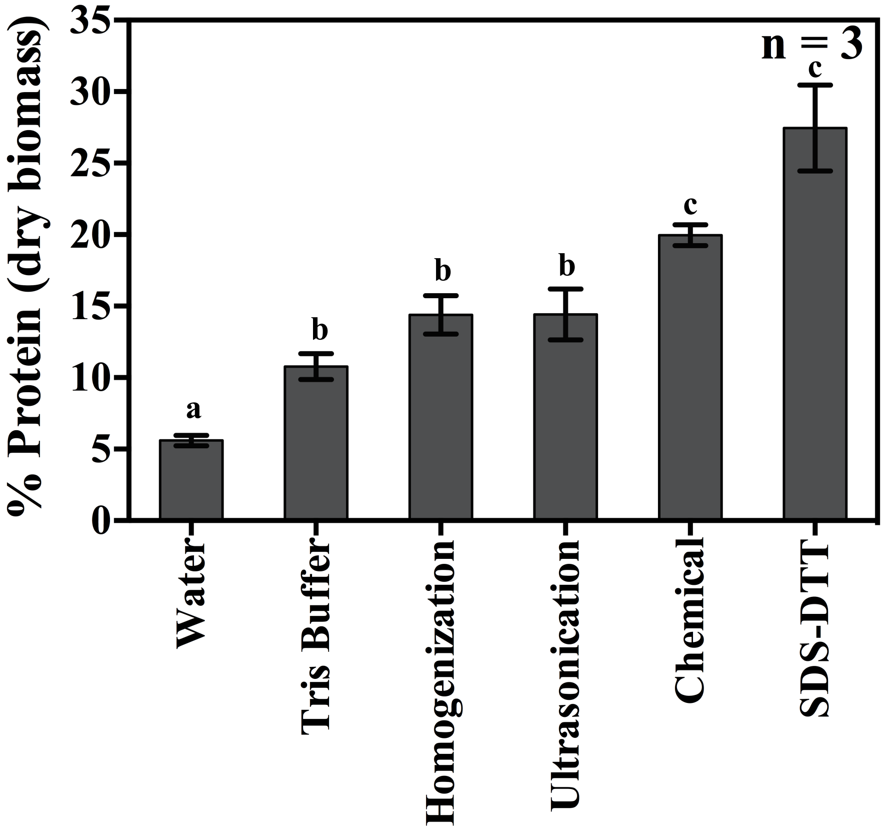

Figure S2: Quantification of proteins by Bradford assay (A). Total proteins were extracted from the marine diatom *Cylindrotheca closterium* using different extraction methodologies and were quantified using BCA as percentage protein of dried biomass. Bovine serum albumin was used as standard for calibration and the absorbance was measured at 562 nm. Measurements were done in triplicates. Lowercase letters indicate the statistical differences.

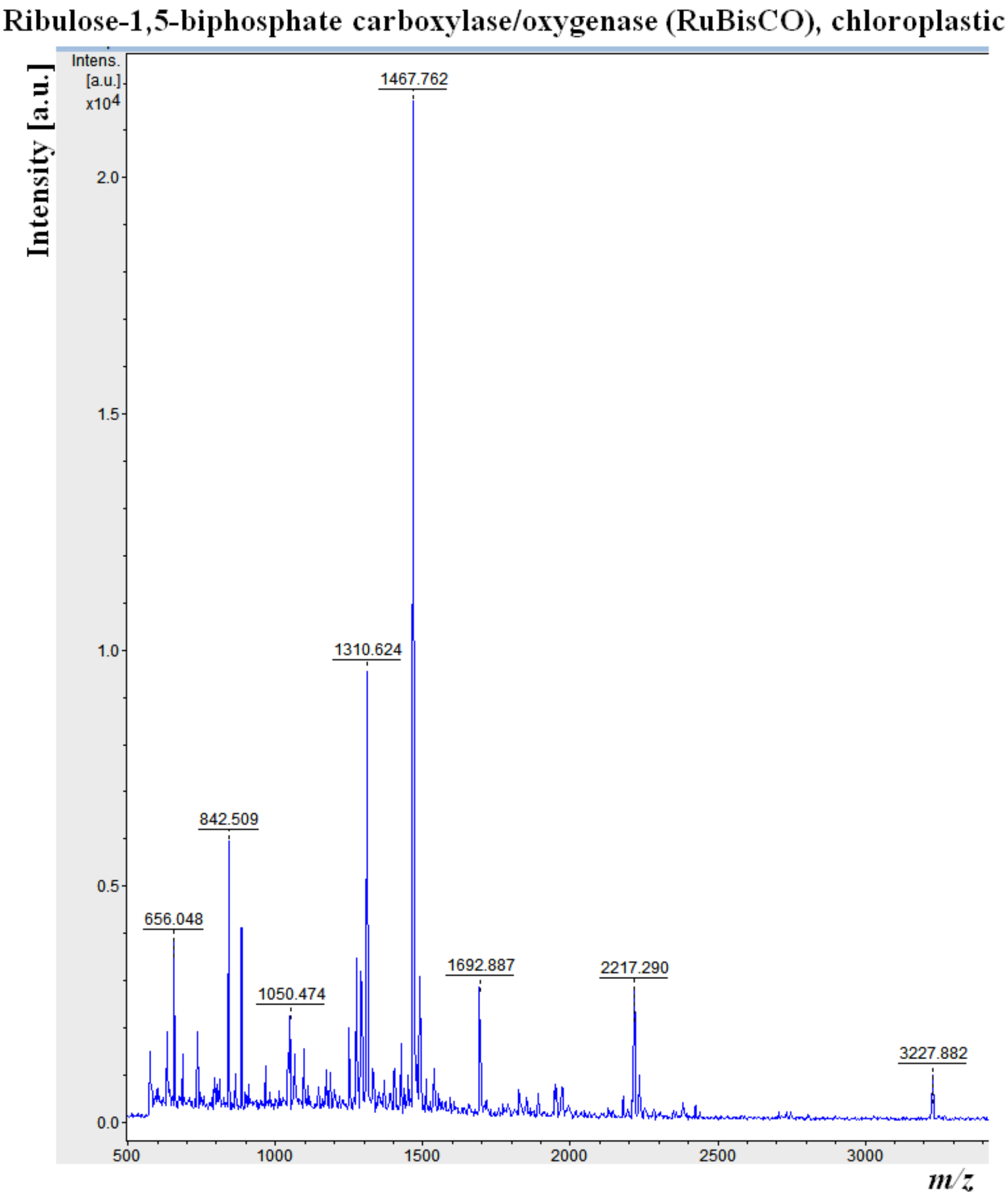

Figure S3: Peptide mass fingerprint mass spectra as observed by MALDI-TOF-MS analysis. MALDI-TOF-MS analysis of tryptically digested *Cylindrotheca closterium* protein spot. All prominent *m/z* ratio peaks from the MALDI MS spectra were subjected to MASCOT analysis and assigned to predicted peptide fragments of ribulose-1,5-biphosphate carboxylase/oxygenase (RuBisCO), chloroplastic.

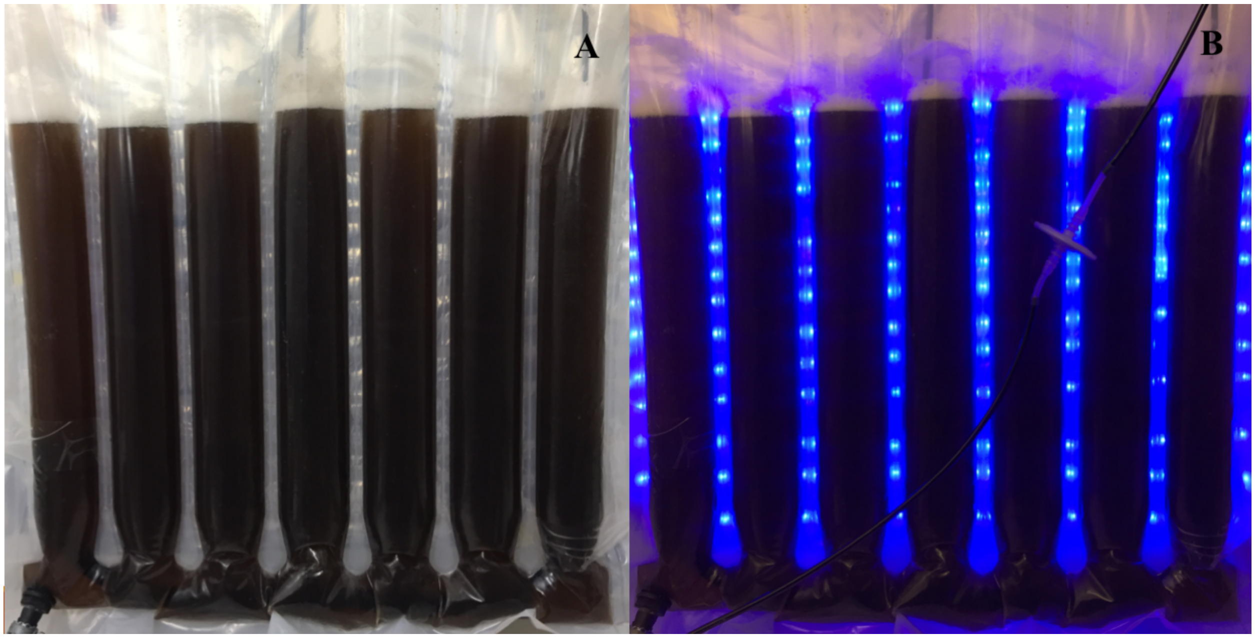

Figure S4: Bag photobioreactors with LED illumination. A: LED panel was off; B: blue LED light was applied to *Cylindrotheca closterium* cultivation.

Table S1: Summarized table for the proteins identified.

Spot ID	Accession No. NCBI	Accession No. UniProt	Annotation	Functional group	Experimental M.W. (kDa)/pI	Theoretical M.W. (kDa)/pI
1	YP_009029066.1	A0A023HC89	Ribulose-1,5-biphosphate carboxylase/oxygenase (RuBisCO), small subunit	Photosynthesis	15/5.1	15.9/5.1
2	YP_009029073.1	A0A023HAY6	ATP synthase subunit b, chloroplastic	Energy Metabolism	17/5.3	20.4/6.9
3	YP_009029070.1	A0A023HAH4	ATP synthase subunit a, chloroplastic	Energy Metabolism	19/4.5	26.9/4.5
4	YP_009029144.1	A0A023HAI9	RuBisCO operon transcription regulator	Photosynthesis	25/4.6	34.9/8.7
6	YP_009029067.1	A0A023HBP6	Ribulose-1,5-biphosphate carboxylase/oxygenase (RuBisCO), large subunit	Photosynthesis	45/4.5	54.0/6.1
7	YP_009029077.1	A0A023HBP8	ATP-dependent zinc metalloprotease FtsH	Cellular Functions	55/5.5	68.4/5.3

Spots, accession numbers (NCBI and Uniprot) and protein names with their functions and experimental M.W. and pI for each of the proteins identified by MALDI-TOF-MS studies.
